# Differentiation enhances Zika virus infection of neuronal brain cells

**DOI:** 10.1038/s41598-018-32400-7

**Published:** 2018-09-28

**Authors:** Claudia Sánchez-San Martín, Tony Li, Jerome Bouquet, Jessica Streithorst, Guixia Yu, Aditi Paranjpe, Charles Y. Chiu

**Affiliations:** 1 Department of Laboratory Medicine, University of California, San Francisco, CA 94107 USA; 2 UCSF-Abbott Viral Diagnostics and Discovery Center, San Francisco, CA 91407 USA; 3 Department of Medicine, Division of Infectious Diseases, University of California, San Francisco, CA 94107 USA

## Abstract

Zika virus (ZIKV) is an emerging, mosquito-borne pathogen associated with a widespread 2015–2016 epidemic in the Western Hemisphere and a proven cause of microcephaly and other fetal brain defects in infants born to infected mothers. ZIKV infections have been also linked to other neurological illnesses in infected adults and children, including Guillain-Barré syndrome (GBS), acute flaccid paralysis (AFP) and meningoencephalitis, but the viral pathophysiology behind those conditions remains poorly understood. Here we investigated ZIKV infectivity in neuroblastoma SH-SY5Y cells, both undifferentiated and following differentiation with retinoic acid. We found that multiple ZIKV strains, representing both the prototype African and contemporary Asian epidemic lineages, were able to replicate in SH-SY5Y cells. Differentiation with resultant expression of mature neuron markers increased infectivity in these cells, and the extent of infectivity correlated with degree of differentiation. New viral particles in infected cells were visualized by electron microscopy and found to be primarily situated inside vesicles; overt damage to the Golgi apparatus was also observed. Enhanced ZIKV infectivity in a neural cell line following differentiation may contribute to viral neuropathogenesis in the developing or mature central nervous system.

## Introduction

Several arthropod-borne flaviviruses are neuropathogenic to humans^1^, including West Nile virus (WNV), Japanese encephalitis virus (JEV), tick-borne encephalitis virus, Powassan virus, and dengue virus^2–4^. Zika virus (ZIKV), transmitted by infected *Aedes* mosquito, is another viral pathogen in the *Flaviviridae* family and was originally discovered in Uganda in 1947. The acute illness caused by ZIKV is typically a self-limited febrile illness characterized by rash, conjunctivitis, and arthralgia. Prior to 2007, there were only 14 reported cases of ZIKV infection in humans^5–9^. A large ZIKV outbreak occurred in Yap Island in the western Pacific Ocean in 2007, during which 73% of the residents on the island were infected^10,11^ Since the Yap outbreak, the rapid and widespread emergence of ZIKV throughout the Western Hemisphere from 2014–2016 has uncovered more severe potential neurological manifestations associated with infection, including cases of Guillain-Barré Syndrome (GBS), meningoencephalitis, and acute flaccid paralysis^12–14^, as well as neurodevelopmental defects such as microcephaly in the infants of infected mothers^15–17^.

A number of *in vitro* and *in vivo* models of ZIKV infection have been developed in the last two years. The susceptibility of human neuronal progenitor cells (NPCs) to ZIKV infection was demonstrated in *in vitro* studies of induced pluripotent stem cells^18^ and with organoids and cortical neurospheres, which recapitulated the cell death, decrease in proliferation, and reduction of organoid volume that was seen in fetal tissues from microcephaly cases^19,20^. These studies demonstrated preferential ZIKV infection of progenitor stem cells, with only a minor percentage of mature neurons affected. Much less is known regarding ZIKV infection of mature, fully differentiated neurons.

Here we sought to establish an *in vitro* model for studying ZIKV infection in undifferentiated and differentiated neural cells of the brain. As a potential model for human adult mature neurons, we used ZIKV to infect the neuroblastoma cell line SH-SY5Y with and without pretreatment with retinoic acid (RA). RA plays different roles in the nervous system, including neural differentiation, axon outgrowth and neuronal patterning. The exact mechanisms of action for RA are still not completely understood, but its effect is associated with the activation of nuclear receptors and induction of different signaling pathways, such as the retinoic receptor and peroxisome proliferator activated-receptor. RA has been shown to promote neuronal differentiation *in vitro* by controlling cell cycle progression^21–23^.

The SH-SY5Y neuroblastoma cell line was originally derived from the cell line SK-N-SH (obtained from a metastatic bone marrow biopsy) as a neuroblast-like subclone after three passages. The subclone was characterized as a N-type neuronal cell line^24^. SH-SY5Y cells have been previously used for the study of several different viruses *in vitro*, including flaviviruses^25–28^. Although both undifferentiated and differentiated SH-SY5Y cells were found to be permissive for ZIKV infection^29,30^, the impact of differentiation on ZIKV infectivity and titers has yet to be fully described.

## Results

**Figure 1:**
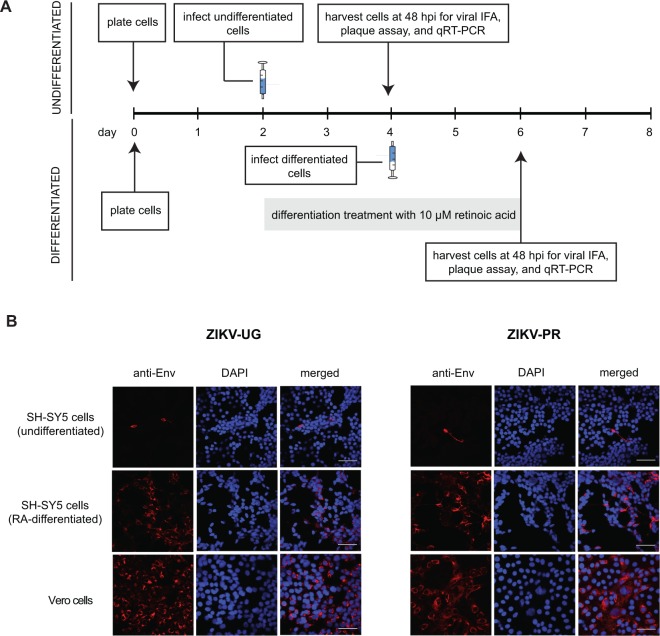
ZIKV infection of SH-SY5Y neuroblastoma cells. (**A**) Infection protocol for undifferentiated cells (top) and cells after 2 days of differentiation with retinoic acid (bottom). Syringe icons denote the day of infection with ZIKV. After one hour of incubation, virus was washed twice and collection of time 0 samples was performed. (**B**) Immunofluorescent staining of SH-SY5Y or Vero cells infected with ZIKV-UG (left) or ZIKV-PR (right) at a multiplicity of infection (MOI) of 1. The 3 columns within each set of 9 panels correspond to staining with a monoclonal antibody against the flaviviral envelope protein (“anti-Env”, staining red, column 1), DAPI stain for cell nuclei (“DAPI”, staining dark blue, column 2), and the merged images (“merged”, column 3). Scale bars represent 50 µm. Abbreviations: IFA, immunofluorescence assay; qRT-PCR, quantitative reverse transcription-polymerase chain reaction; hpi, hours post-infection; DAPI, 4′6-diamidino-2-phenylindole. The images shown are representative images taken from 3 independent experiments, with observation of a minimum of 10 fields under both low (10X) and high (63X) magnification per experiment.

We infected undifferentiated SH-SY5Y cells with the prototype 1947 Uganda strain (MR766) (ZIKV-UG), representing the African lineage, and the contemporary ZIKV strain PRVABC59 (ZIKV-PR), representing the currently circulating Asian lineage (Fig. 1A). At a multiplicity of infection (MOI) of 1, very few cells were positive for ZIKV envelope protein by immunofluorescent staining at 48 hours post-infection (Fig. 1B, top row). Increasing the MOI to 10 did not increase the proportion of infected cells by staining (Supplementary Fig. 1). In contrast, ZIKV infection of control Vero cells, highly permissive to ZIKV infection due to lack of interferon expression, was robust for both the ZIKV-UG and ZIKV-PR strains (Supplementary Fig. 1), and extent of infection was roughly proportional to the input virus concentration (Fig. 1B, bottom row; Supplementary Fig. 1). Interestingly, we observed that many of the infected SH-SY5Y cells showed extensions characteristic of differentiated cells, raising the question of whether differentiated SH-SY5Y cells are more susceptible to ZIKV infection.

To assess this possibility, we evaluated 2 independent established protocols for inducing differentiation of SH-SY5Y cells, incubation in 2% serum or treatment with all-trans-retinoic acid (RA). A treatment dose of 10 µM RA was selected as several studies had previously demonstrated robust differentiation at that concentration^31–33^. Regular SH-SY5Y growing media contained 10% fetal bovine serum. Although both protocols were successful in inducing differentiation, the more effective protocol was the addition of 10 µM RA for 2 days in 10% FBS media (Supplementary Fig. 2), which resulted in extensive production of neurites and formation of a stable monolayer (Supplementary Fig. 3A). To confirm that cells were differentiated, we monitored a number of previously characterized neuronal markers by RNA-Seq transcriptional profiling and immunofluorescent staining (Table 1). Following treatment with RA, there was an increase in the expression of multiple established markers of neuronal differentiation (Table 1 and Supplementary Fig. 3B), including MAP2 (microtubule-associated protein-2), synaptophysin (present at functional synapses), SatB (a transcriptional factor expressed in cortical neurons), GFAP (a marker of astrocytes), and TH (tyrosine hydroxyolase, a dopaminergic marker)^34,35^, as well as corresponding expected decreases in the abundance of vimentin and ID2 genes (Table 1)^36^.

**Table 1:** Neuronal markers expressed in differentiated SH-SY5Y cells.

Marker	log2 fold change (p-value)^a^	IFA signal, undifferentiated^c^	IFA signal (RA-differentiated)^b,c^	predicted effect after differentiation
Synaptophysin	1.96 (4.57E-14)	+	+++	up-regulation
MAP2	1.37 (2.14E-05)	+	+++	up-regulation
ID2	−0.13 (3.68E-86)	ND	ND	down-regulation
vimentin	−0.75 (1.01E-03)	+++	+	down-regulation
SOX2	NSC	−	−	down-regulation
GFAP	NSC	−	−	no effect
SatB	ND	−	+++	up-regulation
TH	ND	−	+++	up-regulation

^a^RA-differentiated versus undifferentiated cells; based on 3 independent replicates.

^b^cells were differentiated with 48 hours of RA treatment.

^c^“−”, no fluorescent signal detected in any cell; “+”, low signal detected in <20% of cells; “+++”, strong signal detected in >70% of cells.

Abbreviations: RA, retinoic acid; SD, standard deviation; IFA, immunofluorescence assay; NEG, negative; POS, positive; MAP2, microtubule-associated protein-2; ID2, inhibitor of DNA binding 2; SOX2, SRY (sex determining region Y)-box 2; GFAP, Glial fibrillary acidic protein; SatB, Special AT-Rich Sequence Binding Protein; and TH, tyrosine hydroxyolase; NSC, not significant change; ND, test not done.

Next, we infected differentiated SH-SY5Y cells (after 2 days of RA treatment) with the ZIKV-UG and ZIKV-PR strains and looked for the expression of viral antigen by immunofluorescent staining at 48 hr post-infection. For both strains, a higher proportion of ZIKV-positive cells (up to 70% higher for ZIKV-UG) was observed by immunofluorescence in differentiated cells versus undifferentiated cells (Fig. 1B**;** Supplementary Fig. 2). These results indicated that pretreatment of SH-SY5Y cells with RA and resultant differentiation rendered these cells more permissive to ZIKV infection. The use of low-serum media (2% FBS) for inducing differentiation also produced an increase in proportion of infected cells to ~60% in ZIKV-UG infected cells (Supplementary Fig. 2).

**Figure 2:**
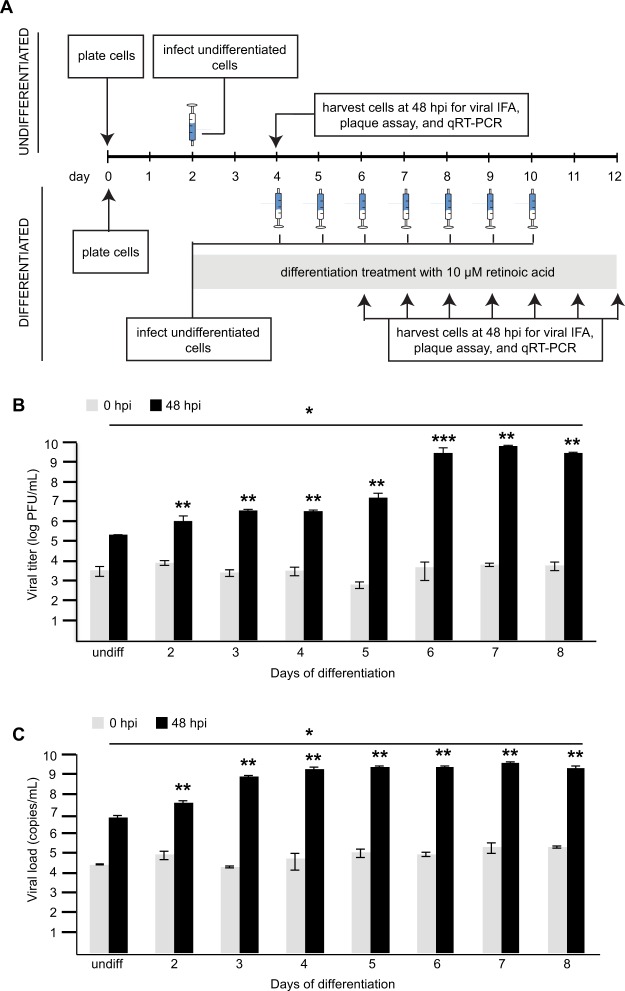
ZIKV infection of SH-SY5Y neuroblastoma cells following longer periods of retinoic acid-induced differentiation. (**A**) Infection protocol for undifferentiated cells (top) and cells after 2 to 8 days of differentiation with retinoic acid (bottom). Syringe icons denote the day of infection with ZIKV-UG. After one hour of incubation, virus was washed twice and collection of time 0 samples was performed. Infection was monitored after 48 hours by IFA, plaque assay and qRT-PCR. (**B**) Plot of viral titers as determined by plaque assay versus days of differentiation prior to ZIKV infection. (**C**) Plot of viral titers as determined by qRT-PCR at different days of differentiation. Gray bars represent control data from 0 hours post infection (hpi), and black bars represent data from 48 hpi. Titers and error bars are calculated from the average and standard deviation of three independent experiments. *p < 0.05 by analysis of variance (ANOVA); **p < 0.05 by one-tailed t-test; ***p = 0.07 by one-tailed t-test. Abbreviations: IFA, immunofluorescence assay; qRT-PCR, quantitative reverse transcription-polymerase chain reaction; hpi, hours post-infection; PFU, plaque-forming units.

To determine whether mature differentiated cells were being preferentially infected by ZIKV in RA-treated SH-SY5Y cells, we co-stained these cells with immunofluorescent antibodies targeting the flaviviral envelope protein (anti-Env) and the neural differentiation marker TH (Supplementary Fig. 4). Most, but not all, of the infected cells positive for ZIKV using the anti-Env antibody were also positive with TH staining (Supplementary Fig. 4). This showed that although ZIKV-UG was able to infect both differentiated and undifferentiated cells, the proportion of infected cells was higher in those differentiated or undergoing differentiation. We then tested whether extended exposure to RA to induce more differentiation resulted in further increases in ZIKV-UG infection. SH-SY5Y cells were treated with RA for up to 8 days prior to viral infection (Fig. 2A) and infectious viral titers as a function of days of differentiation determined by plaque assay (Fig. 2B). RA treatment of 2 to 5 days in duration produced similar increases in infection, whereas extended treatment of 6 to 8 days resulted in a further 2-log increase in viral titers.

To investigate the hypothesis that decreases in the relative expression of interferon-associated genes may explain increased susceptibility of RA-differentiated versus undifferentiated SH-SY5Y cells to ZIKV infection, we analyzed the transcriptome profiling data for levels of expression of interferon-stimulated genes (ISGs). We did not observe down-regulation of ISGs in differentiated cells; rather, genes associated with type I interferon signaling, such as IRF9 and STAT2, were up-regulated in RA-differentiated cells, and the only significantly down-regulated gene identified, DSCT1, was a negative regulator of type I interferon signaling^37^. This suggested that the enhanced ZIKV infection in RA-differentiated cells was not due to basal decreases in the expression of ISGs.

The Axl protein has been postulated as a putative cell entry receptor for ZIKV. To determine whether the observed enhanced infection in differentiated cells following 2 days of RA treatment was due to increased expression of AxI, we stained differentiated and undifferentiated SH-SY5Y cells (both uninfected and after 48 hours infection by ZIKV-UG or ZIKV-PR) with an Axl-specific monoclonal antibody, which detects endogenous levels of total Axl. Under all tested conditions, the Axl immunofluorescent signal was at the noise level (Supplementary Fig. 5), similar to that using only the secondary antibody as a negative control. Consistent with this observation, analysis of Axl RNA by RNA-Seq transcriptome analysis showed basal levels of expression, which were not increased after RA treatment; the FPKM (fragments per kilobase of transcript per million mapped reads) value was 0.0072 in undifferentiated cells and 0.0063 in RA treated cells, indicating a non-significant log2 fold change of −0.19 (using a log2 fold change of ≥1.5 as a cutoff for significance)^38^. This indicated that Axl was not expressed at a detectable level in either differentiated or undifferentiated SH-SY5Y cells.

**Figure 3:**
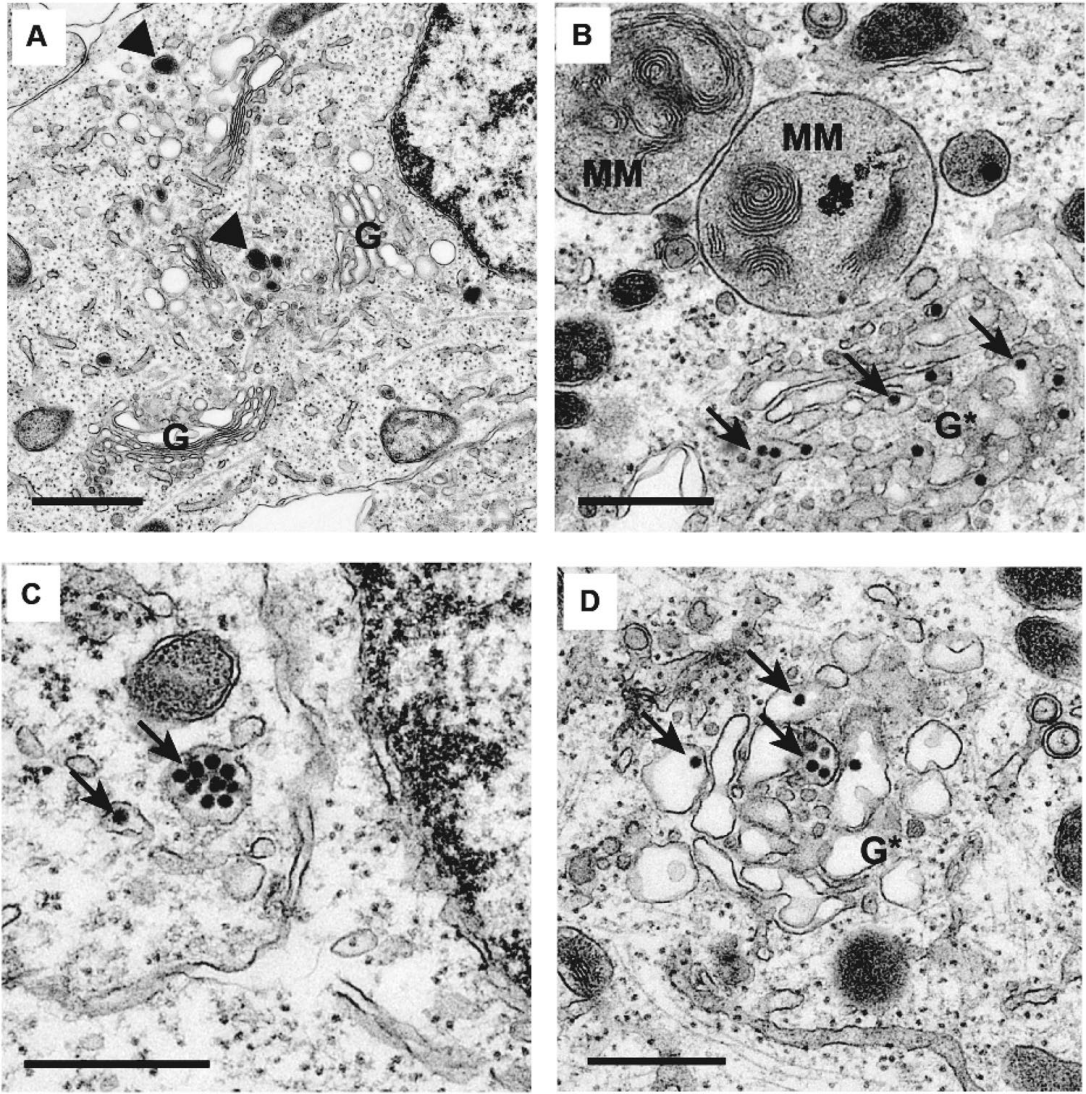
Transmission electron microscopy of uninfected and ZIKV-infected differentiated SH-SY5Y cells. (**A**) Uninfected control. Arrowheads (black) show typical neurotransmitter vesicles. (**B–D**) Differentiated SH-SY5Y cells at 48 hours post-infection with ZIKV-UG. Arrows (black) point to representative viral particles. G, intact Golgi complex; G*, disrupted Golgi complex; MM, multi-membranous areas. Cells are treated with 2 days of retinoic acid prior to ZIKV infection. The scale bar represents 0.5 µm.

**Figure 4:**
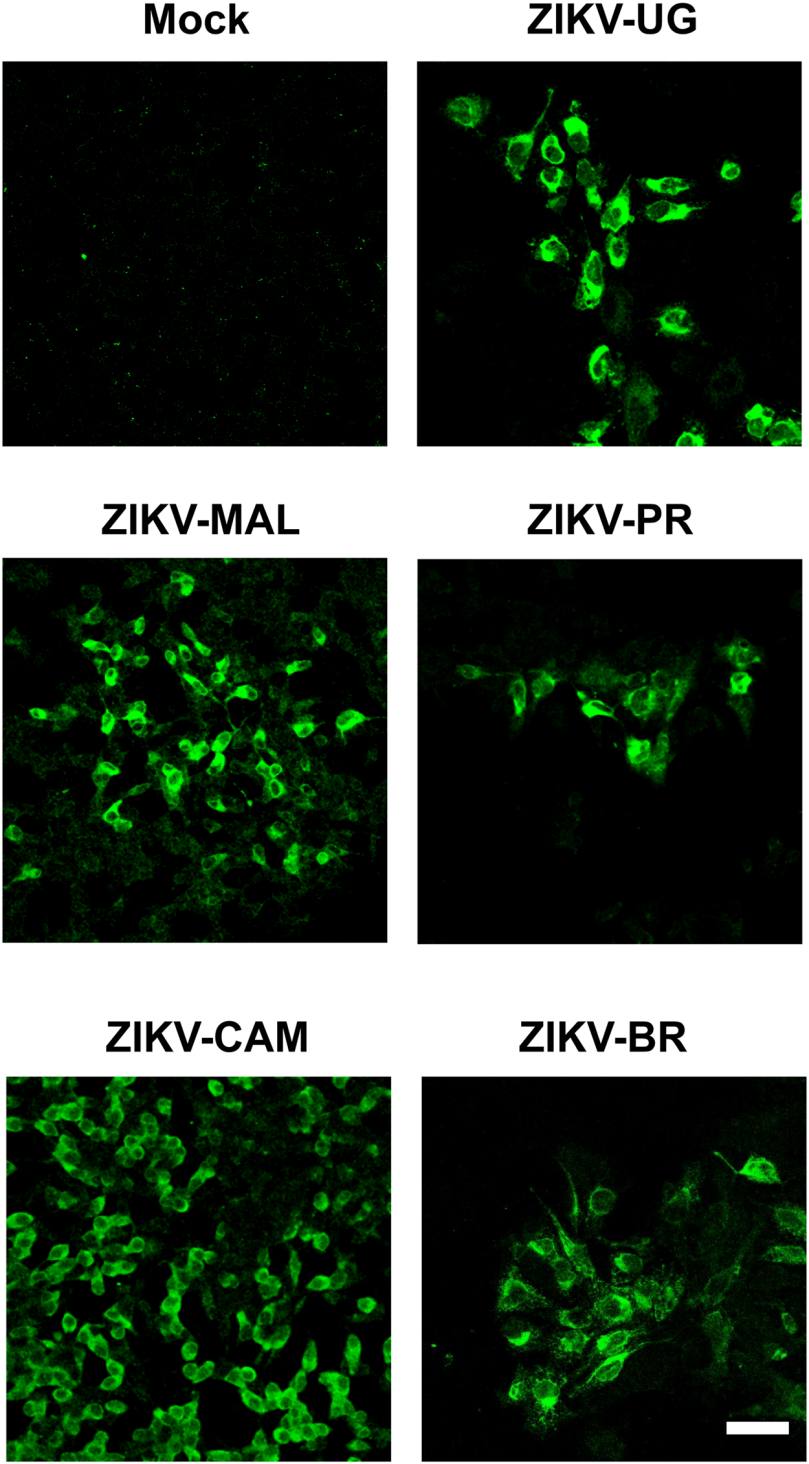
Infection of differentiated SH-SY5Y cells with ZIKV strains representing African and multiple Asian lineages. Viral infection is monitored by immunofluorescent staining of differentiated SH-SY5Y cells at 48 hours post-infection using the anti-Env monoclonal antibody (green). Cells are treated with 2 days of retinoic acid prior to ZIKV infection. The images shown are representative images taken from 3 independent experiments, with observation of a minimum of 10 fields under both low (10X) and high (40X and 63X) magnification per experiment. The scale bar represents 50 µm.

Next, we infected differentiated SH-SY5Y cells after 48 hours of RA treatment with mock supernatant (Fig. 3A) or ZIKV-UG (Fig. 3B–D), and fixed and processed the samples at 48 hr post-infection for visualization by transmission electron microscopy. In ZIKV-UG infected cells, we observed clusters of high-density spherical viral particles ~50 nm in diameter inside cytoplasmic vesicles and the Golgi apparatus (Fig. 3B–D). Overt distortion and membrane disruption of the Golgi complex were observed in multiple areas (Fig. 3B,C). Differentiated SH-SY5Y cells were also infectable by 3 additional ZIKV strains in the Asian lineage: the Malaysian strain P6740 (ZIKV-MAL), the Cambodian strain FSS13025 (ZIKV-CAM) and the Brazil strain SPH2015 (ZIKV-BR) (Fig. 4).

## Discussion

In this study, we report productive infection of neuroblastoma SH-SY5Y cells by multiple ZIKV strains from the African and Asian lineages. Although the African ZIKV-UG strain has been passed extensively in mouse brain^39^, all of the contemporary, low-passage Asian strains were able to infect these cells. We also present evidence that differentiated SH-SY5Y cells resembling mature neurons are more efficiently infected by ZIKV than undifferentiated cells. Increased susceptibility of neural SH-SY5Y cells to ZIKV infection with degree of differentiation suggests a role for enhanced viral infection in ZIKV-associated neuroinvasive diseases in both adults and children, including acute flaccid paralysis (AFP) and meningoencephalitis. Enhanced viral infection in the setting of actively differentiating neurons in the developing brain may be a contributing factor in ZIKV-associated fetal neurodevelopmental disorders such as microcephaly

Successful differentiation of SH-SY5Y cells after two days of RA treatment was documented by the presence of neural extensions and expression of cell markers characteristic of mature neurons. Cells differentiated using two independent methods were more susceptible to ZIKV infection than undifferentiated cells, as evidenced by a higher proportion of infected cells by immunofluorescence and infectious viral titers in the supernatant. These findings contrast with those of a previous study showing that partially differentiated SH-SY5Y neuroblastoma cells were not more susceptible to infection than undifferentiated cells, and that two terminally differentiated olfactory bulb neuroblastoma cell lines were reported as resistant to ZIKV infection^29^. The discrepancy in these observations is likely due to differences in experimental design. In the aforementioned report, SH-SY5Y cells were cultured up to 7 days and then infected with ZIKV, whereas in our experiments cells were infected soon after seeding or after 2 days of RA treatment. Specifically, we observed that culturing of uninfected SH-SY5Y cells for >3 days resulted in the production of neurite extensions, even in standard 10% FBS media. Another difference that may account for the discrepant results between the prior study and ours may be the approach used for differentiation: here we used 10 µM RA for differentiation, as done previously^32^, and confirmed differentiation by assaying for the presence of mature neuronal markers, whereas only 1 µM RA was used in the previous study^29^.

Some potential explanations for the observed enhancement of ZIKV infection following differentiation are down-regulation of antiviral responses or different receptor usage in differentiated relative to undifferentiated cells. However, we found by transcriptome profiling that the overall basal expression of ISGs in RA-differentiated SH-SY5Y cells was increased, and not decreased, relative to undifferentiated cells. In addition, we did not detect evidence of increased expression of Axl, proposed as a candidate receptor for ZIKV^40^. Multiple lines of evidence from other published reports corroborate our finding of low or absent baseline Axl expression in SH-SY5Y cells, indicating that Axl is not necessary for ZIKV infection in this neural cell line. First, Axl expression has not been detected in other neuroblastoma cell lines such as JMR32 and SK-N-SH^41,42^. Second, the use of alternative receptors to Axl for ZIKV entry in neuronal cells has been demonstrated, as infection of the eye and optic nerve by ZIKV in adult mice was found to be Axl-independent^43^. Third, Axl signaling is not required for productive ZIKV replication in human astrocytes^44^, nor for infection of human and murine brain organoids or mice lacking Axl protein^20,43^. Further investigation will be needed to determine the underlying mechanism(s) for the increased susceptibility of differentiated SH-SY5Y neuroblastoma cells to ZIKV infection.

## Materials and Methods

### Cell lines and reagents

Human neuroblastoma cells SH-SY5Y (ATCC CRL-2266) and African green monkey kidney VERO E6 cells (ATCC CRL1586) were obtained from the American Type Culture Collection (ATCC, Manassas, VA). SH-SY5Y were cultured in 1:1 Eagle’s Minimum Essential Media (EMEM) and Ham’s F12 supplemented with 10% v/v fetal bovine serum (FBS), 1% penicillin-streptomycin, and 2 mM glutamine (Sigma-Aldrich, Carlsbad, CA). Cells were maintained in a logarithmic phase of growth in 25 mL or 75 mL plastic culture flasks (BD Falcon). SH-SY5Y cells were differentiated by treatment with 10 µM retinoic acid (RA) (Life Technologies). All cells were maintained at 37 °C in a humidified incubator with a 5% CO2 atmosphere.

ZIKV strain PRVABC59 (ZIKV-PR) was obtained from the Centers for Disease Control and Prevention (CDC) (GenBank accession number KU501215), and passed twice in Vero cells. This strain was originally isolated in 2015 from the serum of a patient who traveled to Puerto Rico, and had been passaged three times in Vero cells. The prototype African ZIKV strain MR766 ZIKV-UG) was provided by the California Department of Public Health (Richmond, CA); this strain was isolated in Uganda in 1947, and has since been passed 147 times in mice brain and 3 times in Vero cells. The Cambodian strain FSS13025 (ZIKV-CAM) was isolated in 2010 and provided by Joseph DeRisi at University of California, San Francisco (UCSF) (San Francisco, CA). The Brazil strain SPH2015 (ZIKV-BR) was isolated in 2015 (GenBank accession number KU321639)^45^, and was provided by Marion Lanteri at the Blood Systems Research Institute (San Francisco, CA). The Malaysian strain P6740 (ZIKV-MAL) was provided by Raul Andino at UCSF. The last 3 aforementioned strains were propagated in Vero cells infected at an MOI of 0.01 and kept at low passage numbers. ZIKV supernatants used for infection experiments were collected at 96 or 120 hours post-infection (hpi) and clarified by centrifugation at 350 × g for 30 min. The ZIKV-UG, ZIKV-PR and ZIKV-BR cultures were sequenced on a MiSeq instrument at >10X redundancy and no differences between the assembled consensus sequence and GenBank reference sequence were found. Flavivirus group envelope mouse monoclonal antibody (mAb) (anti-Env), which cross-reacts with ZIKV and other flaviviruses such as dengue virus, was obtained from EMD Millipore (catalog number MAB10216, clone D1-4G2-4-15). For Axl detection, rabbit mAb C89E7 (Cell Signaling Technology, Danvers, MA) was used.

### Virus infection

SH-SY5Y cells were plated in 24-well plates at a concentration of 2 × 10^5^ per well and infected with different ZIKV strains at a multiplicity of infection (MOI) of 0.1. 1 or 10, for one hour, followed by washing twice, and then kept in MEM and 1% FBS media.

### Immunofluorescence

Differentiated or undifferentiated cells were infected with ZIKV, fixed with 4% paraformaldehyde for 10 min, washed twice with 0.2% bovine serum albumin and phosphate-buffered saline (BSA/PBS), and incubated with primary antibody for 30–60 min at room temperature. Next, cells were washed twice with 0.2% BSA/PBS, incubated with secondary antibody for 30 min, and finally washed twice again with 0.2% BSA/PBS. Cells were permeabilized with 0.1% Triton X-100 (Thermo-Fisher Scientific) for analysis of internal epitopes, or fixed directly with cold methanol for 10 min. Stained cells were analyzed using a Nikon C1si or Ti confocal microscopes.

### Measurement of infectious ZIKV titers by plaque assay

Vero cells were plated in 6-well plates at a concentration of 3 × 10^5^ per well. After 2 days of growth, 500 µL serial dilutions of ZIKV-infected supernatant from 10^−2^ to 10^−7^ were added to duplicate wells of 6-well plates, followed by incubation at 37 °C for 1 hr for adsorption. After adsorption, the inoculum was removed from each well and overlaid with 2X Eagle’s Minimum Essential Medium with 1% FBS, 1% penicillin-streptomycin and 2 mM glutamine, and 1% of carboxymethyl cellulose in PBS in a 1:1 ratio. Plates were maintained at 37 °C, 5% CO2 for five days, followed by fixation and staining of the cells with 2 mL of crystal violet solution (0.25% crystal violet, 10% methanol, 1.75% formaldehyde, 0.5% CaCl and 35 mM of Tris Base) per well. After 15–20 min incubation and rinsing with tap water, plaques were counted and calculated in plaque forming units per mL (PFU/mL). Statistical analysis was performed with GraphPad Prism software (version 7).

### RNA-Seq transcriptome profiling

Total RNA from cell culture lysates (1 million cells/sample) was extracted using the Direct-zol RNA MiniPrep Kit (Zymo Research). The Ovation Human Blood RNA-Seq Kit (NuGEN Technologies Inc., San Carlos, CA) was used to generate strand-specific RNA-seq libraries. Libraries were sequenced as 100 base pair (bp) paired-end runs on a HiSeq. 2500 instrument (Illumina, San Diego, CA). Paired-end reads were mapped to the human genome (hg19) using STAR v3.12.2^46^, gene and transcript counts were calculated by HTSeq v0.60^47^. Differential expression of genes was performed using EdgeR v3.12.2^48^ implemented in the R programming language. Genes with fold change >±2, *p*-value < 0.05, and adjusted *p*-value (or false discovery rate, FDR) < 0.1% are considered to be differentially expressed. Pathway and network analyses of the transcriptome data were performed using Ingenuity Pathway Analysis software (Qiagen, Valencia, CA). In order to identify the DEGs related to interferon pathways, we retrieved the complete list of interferon pathways genes using the PANTHER database^49^.

### RNA extraction and quantitative reverse transcription polymerase chain reaction (qRT-PCR)

Total RNA from cell culture lysates and corresponding supernatants was extracted using the Direct-zol RNA MiniPrep Kit (Zymo Research, Tustin, CA). ZIKV RNA loads were determined by qRT-PCR testing using primers targeting the envelope gene (ZIKV-1086-ZIKV-1162)^10^.

### Electron microscopy

Cells were collected at 48 hours post infection and fixed in 2% glutaraldehyde, 1% paraformaldehyde in 0.1 M sodium cacodylate buffer pH 7.4, followed by post-fixation in 2% osmium tetroxide in the same buffer, staining with 2% aqueous uranyl acetate, dehydration in acetone, and embedding in LX-112 resin (Ladd Research Industries, Burlington, VT). Samples were sectioned on a Reichert Ultracut S ultramicrotome and counterstained with 0.8% lead citrate. Grids were examined on a JEOL JEM-1230 transmission electron microscope (JEOL USA, Inc., Peabody, MA) and photographed with the Gatan Ultrascan 1000 digital camera (Gatan Inc., Warrendale, PA).

### Accession numbers

RNA-seq data from transcriptome profiling of SH-SY5Y cells have been submitted to the National Center for Biotechnology Information (NCBI) Gene Expression Omnibus (GEO) repository (BioProject accession number PRJNA234047).

## Electronic supplementary material

Supplementary Information
