# Supplementary material for: Differentiation enhances Zika virus infection of neuronal brain cells
Source: Sci Rep. 2018 Sep 28;8:14543. doi: 10.1038/s41598-018-32400-7 (PMC6162312; doi:10.1038/s41598-018-32400-7)
Supplement: Supplementary file 1 — Supplementary Information [file 41598_2018_32400_MOESM1_ESM.pdf]

Differentiation enhances Zika virus infection of neuronal brain cells

Claudia Sanchez San Martin[1,2], Tony Li[1,2], Jerome Bouquet[1,2], Jessica Streithorst[1,2], Guixia Yu[1,2], Aditi Paranjpe[1,2], and Charles Chiu[1,2,3*].

[1]Department of Laboratory Medicine, University of California, San Francisco, CA 94107, USA.

[2]UCSF-Abbott Viral Diagnostics and Discovery Center, San Francisco, CA 91407, USA.

[3]Department of Medicine, Division of Infectious Diseases, University of California, San Francisco, CA 94107

Table S1. Differential expression of interferon-stimulated genes (ISGs) from retinoic acid (RA)-differentiated neuroblastoma SH-SY5Y cells relative to undifferentiated cells.

ISG[a]	log2 fold change[b]	P-value	false discovery rate
ABCE1	-0.531	1.04E-04	1.79E-03
ACTG1	-0.957	1.51E-11	1.58E-09
ADAMTS13	0.699	2.10E-04	3.17E-03
B2M	-0.736	1.24E-05	2.99E-04
BTN3A2	0.501	1.44E-02	9.31E-02
C1QBP	-0.770	4.04E-10	3.18E-08
CCL2	3.549	7.27E-21	2.60E-18
CEBPG	-0.519	5.11E-04	6.56E-03
CX3CL1	-0.673	2.73E-06	8.24E-05
DCST1	-2.690	1.21E-02	8.19E-02
DTX4	1.636	1.19E-04	1.99E-03
EDN1	3.703	1.52E-02	9.72E-02
EGR1	0.769	7.36E-04	8.84E-03
EPRS	-0.531	1.09E-05	2.68E-04
EVL	-0.860	1.51E-16	3.14E-14
GAPDH	-0.537	2.89E-04	4.15E-03
GATA3	-0.936	1.00E-11	1.06E-09
GSN	0.706	7.89E-04	9.33E-03
HMGB2	-0.528	1.53E-06	5.02E-05
HPX	2.207	9.28E-03	6.71E-02
HRAS	-0.501	3.86E-03	3.41E-02
ICAM1	4.339	2.15E-03	2.10E-02
IFI27L2	0.849	8.26E-10	6.18E-08
IFI6	1.499	7.15E-11	6.55E-09
IFITM3	4.493	1.65E-03	1.70E-02
IFNAR2	0.584	3.71E-03	3.29E-02
IFNGR1	-0.586	4.90E-05	9.48E-04
IFRD1	-0.898	1.22E-13	1.76E-11
IL20RA	-0.663	1.06E-02	7.40E-02
INHBA	0.593	7.83E-03	5.91E-02
IRF1	1.483	1.63E-03	1.69E-02
IRF2BP1	-0.975	6.72E-14	1.00E-11
IRF2BP2	-0.561	6.14E-06	1.66E-04
IRF3	0.705	2.39E-06	7.39E-05
IRF9	1.098	2.22E-06	6.95E-05
MYD88	0.587	1.44E-04	2.32E-03
MYO1C	0.597	1.30E-07	5.76E-06
NFKB1	-0.621	2.62E-04	3.81E-03
OAS1	1.106	1.07E-02	7.45E-02
PCBP2	-0.848	5.14E-14	7.73E-12

PDE12	-0.598	8.77E-05	1.55E-03
PIN1	0.558	4.45E-05	8.74E-04
PML	-0.781	4.40E-09	2.84E-07
PPARG	3.703	1.52E-02	9.72E-02
PQBP1	-0.645	3.03E-06	8.98E-05
PRKCD	-0.640	7.89E-03	5.93E-02
PRKDC	-0.591	1.44E-08	8.25E-07
RELA	-0.611	2.94E-08	1.53E-06
RNF26	-0.526	3.26E-05	6.71E-04
SHMT2	-1.108	8.80E-11	7.80E-09
SNCA	0.749	8.49E-06	2.16E-04
STAT2	0.600	2.03E-05	4.51E-04
STXBP3	0.601	5.45E-03	4.45E-02
TP53	-1.116	8.83E-26	5.09E-23
TREX1	1.033	6.46E-04	7.94E-03
TRIM68	0.578	1.08E-02	7.50E-02
TXK	2.880	2.28E-05	4.97E-04
ZC3H12A	1.242	1.52E-02	9.69E-02
ZFPM1	-1.315	1.56E-19	4.50E-17
ZP3	0.793	9.91E-03	7.04E-02
ZYX	-0.582	1.09E-04	1.84E-03

[a] ISGs with absolute log2 fold change of >0.5 are shown.

[b] Significantly upregulated genes (log2 fold change > 1.5) are highlighted in pink. Only one gene, DCST1, a negative regulatory of type I interferon signaling (Nair, et al., *Scientific Reports*, 6:36179, 2016), was found to be significantly down-regulated (log2 fold change < -1.5) in RA-differentiated versus undifferentiated cells.

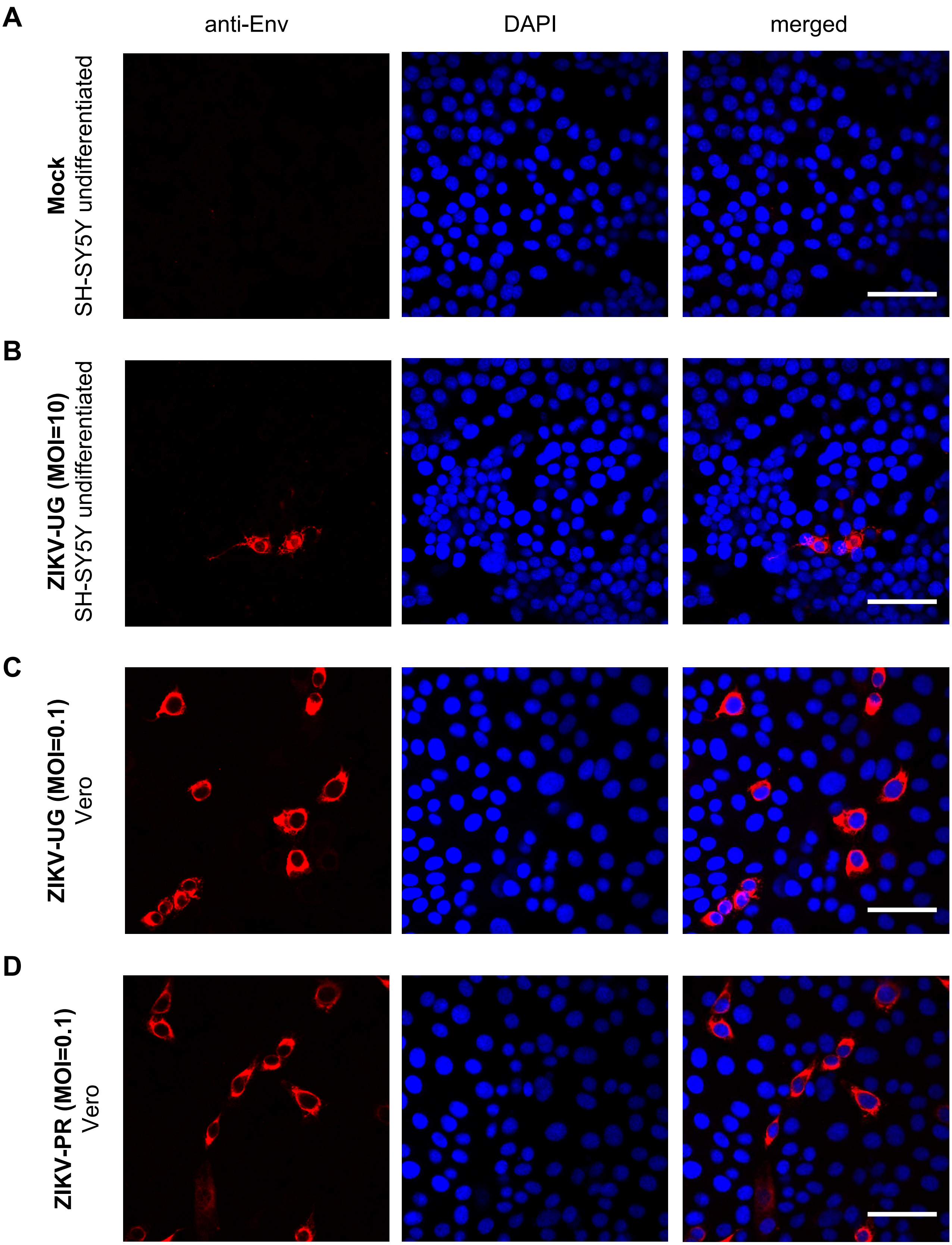
A
B
C
D
anti-Env
DAPI
merged
Mock
SH-SY5Y undifferentiated
ZIKV-UG (MOI=10)
SH-SY5Y undifferentiated
ZIKV-UG (MOI=0.1)
Vero
ZIKV-PR (MOI=0.1)
Vero

Figure S1. ZIKV infection of undifferentiated SH-SY5Y and Vero cells. (A) Mock infection. (B) Undifferentiated SH-SY5Y cells infected with ZIKV-UG at a MOI of 10. (C) Vero cells infected with ZIKV-UG at a MOI of 0.1. (D) Vero cells infected with ZIKV-PR at a MOI of 0.1. Cells are stained with a monoclonal antibody against the flaviviral envelope protein (“anti-Env”, staining red, leftmost column) or DAPI stain for cell nuclei (“DAPI”, staining dark blue, middle column). The rightmost column shows the merged images (“merged”). Scale bars represent 50 µm. The images shown are representative images taken from 3 independent experiments, with observation of a minimum of 10 fields under both low (10X) and high (40X and 63X) magnification per experiment.

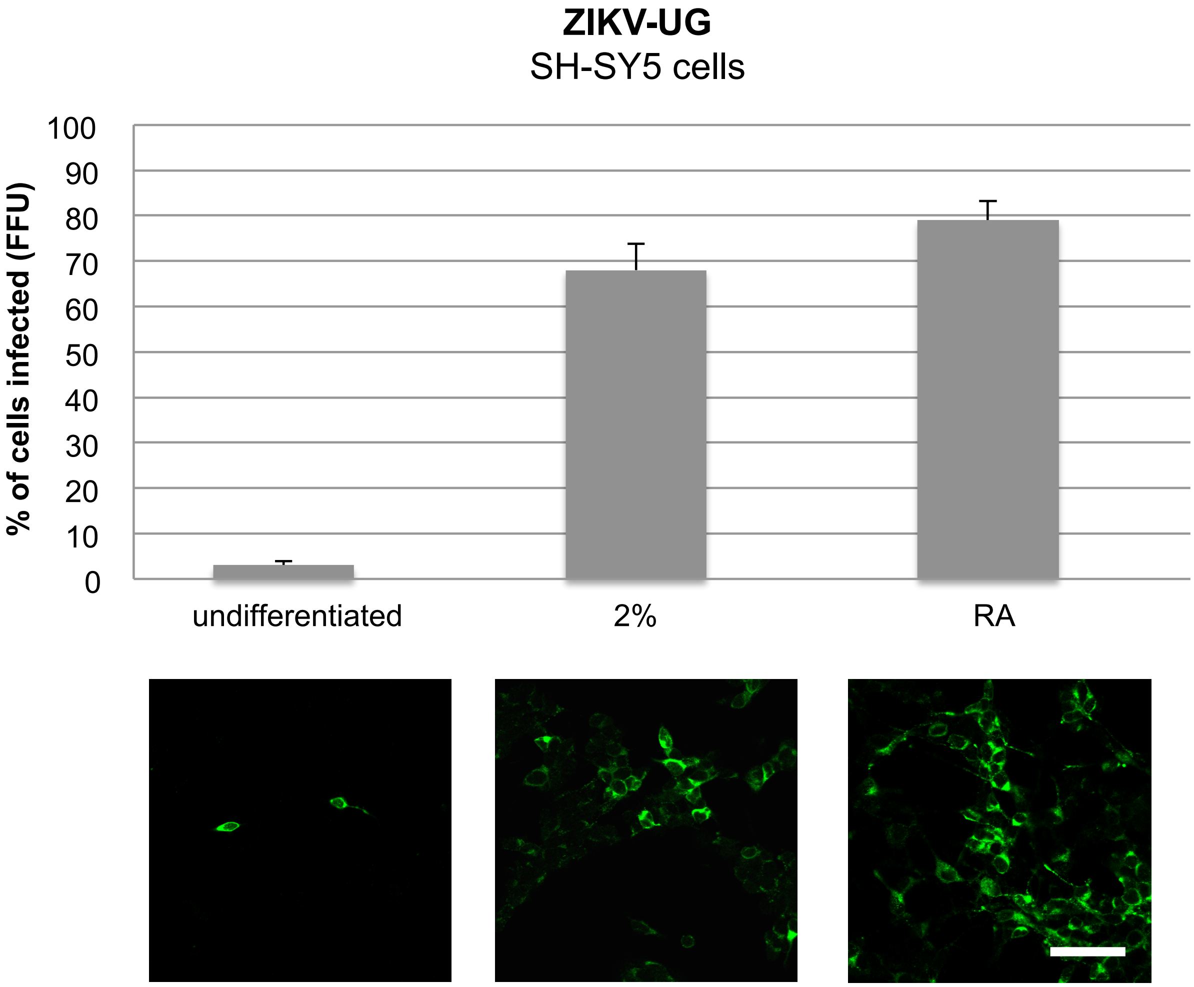

Figure S2. Comparison of methods for differentiation of SH-SY5Y cells. Undifferentiated cells ("undifferentiated") and cells differentiated using low serum treatment with 2% FBS ("2%") or retinoic acid treatment ("RA") for 2 days, were infected with ZIKV-UG at a MOI of 1, followed by harvesting for immunofluorescent staining at 48 hours post-infection (hpi). Shown is a bar graph of the percentage of infected cells staining positive with the anti-Env mAb (out of ~500 cells counted), along with a representative field of view corresponding to each method. The scale bar represents 50 μm. The images shown are representative images taken from 3 independent experiments, with observation of a minimum of 10 fields under both low (10X) and high (40X and 63X) magnification per experiment.

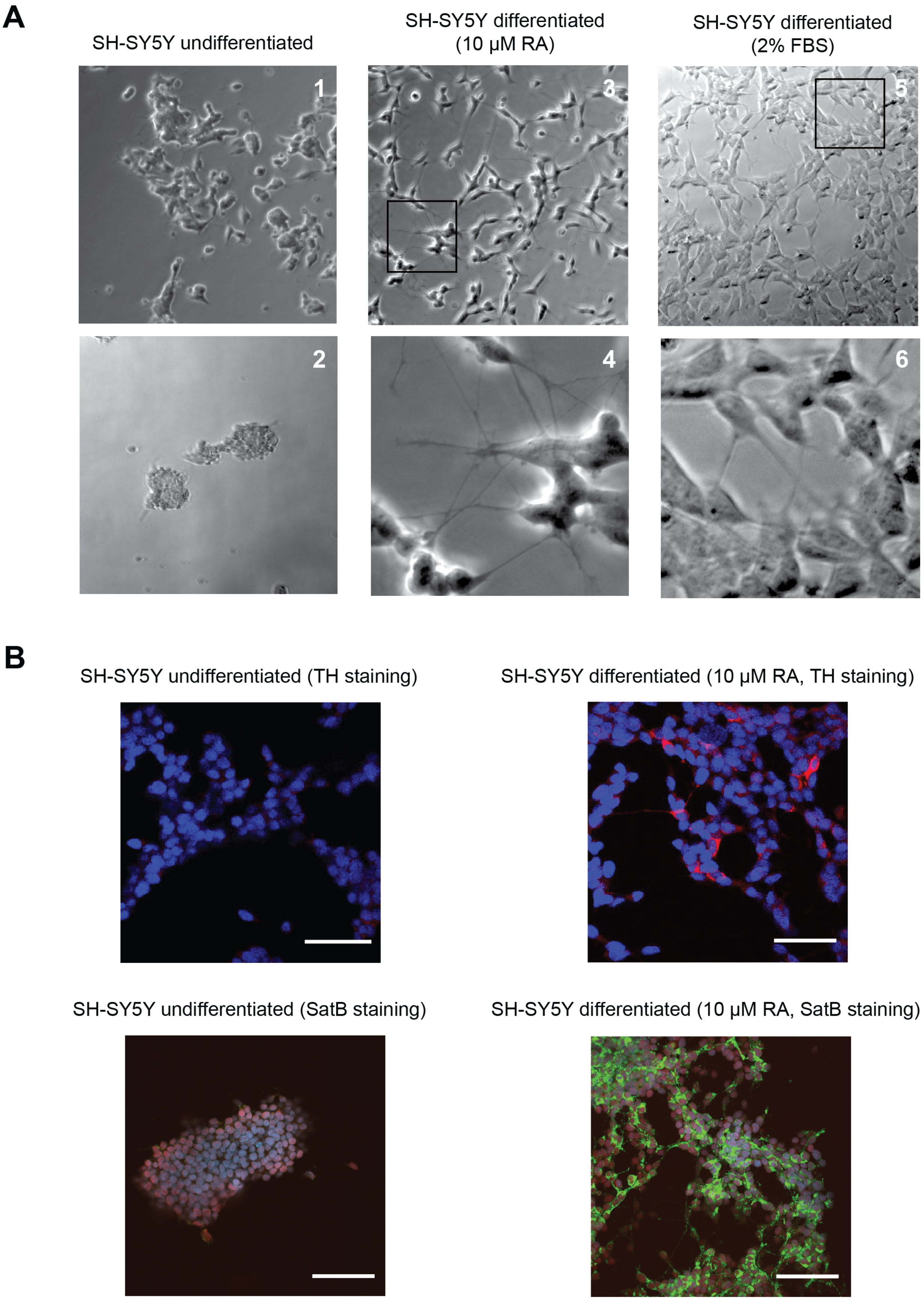
A
SH-SY5Y undifferentiated
SH-SY5Y differentiated (10 µM RA)
SH-SY5Y differentiated (2% FBS)
1
2
3
4
5
6
B
SH-SY5Y undifferentiated (TH staining)
SH-SY5Y differentiated (10 µM RA, TH staining)
SH-SY5Y undifferentiated (SatB staining)
SH-SY5Y differentiated (10 µM RA, SatB staining)

Figure S3. Production of neurites and expression of neural markers SatB and tyrosine hydroxylase (TH) in undifferentiated and differentiated SH-SY5Y cells. (A) Cellular morphology of SH-SY5Y cells. Undifferentiated cells show two morphologies: dispersed cells (panel 1) or clusters of cells (panel 2). Neurites are observed after 2 days of differentiation with either 10 μM retinoic acid (RA) (panel 3 and zoomed inset at 4X magnification in panel 4) or 2% FBS (panel 5 and zoomed inset at 4X magnification in panel 6) treatment. **(B)** Immunofluoreascent staining of differentiated SH-SY5Y cells after 2 days of treatment with RA. Staining for TH protein is shown in red, staining for SatB protein in green, and DAPI nuclear staining in dark blue for TH, and in light red for SatB. The images shown are representative images taken from 3 independent experiments, with observation of a minimum of 10 fields under both low (10X) and high (40X and 63X) magnification per experiment. Scale bars represent 50 μm.

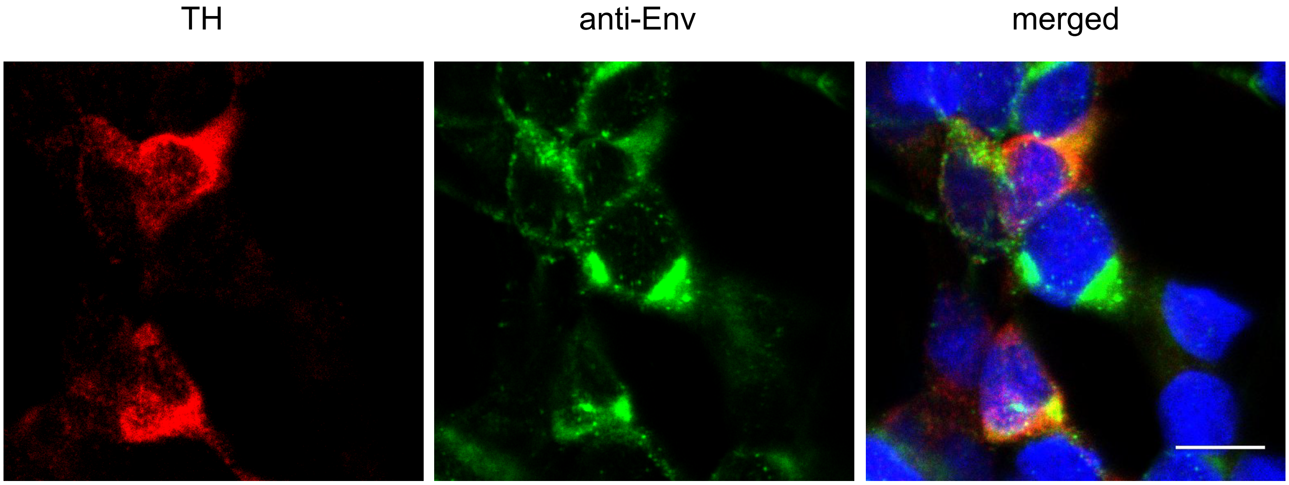

Figure S4. Co-localization of ZIKV infection with neural marker tyrosine hydroxylase (TH) in differentiated SH-SY5Y cells. Cells were treated with retinoic acid for 2 days and infected with ZIKV-UG, followed by fixation and staining at 48 hpi. Staining for TH protein is shown in red (“TH”), ZIKV using the anti-Env antibody in green (“anti-Env”). The merged image including DAPI nuclear staining in dark blue is shown in the rightmost panel. The images shown are representative images taken from 3 independent experiments, with observation of a minimum of 10 fields under both low (10X) and high (40X and 63X) magnification per experiment.

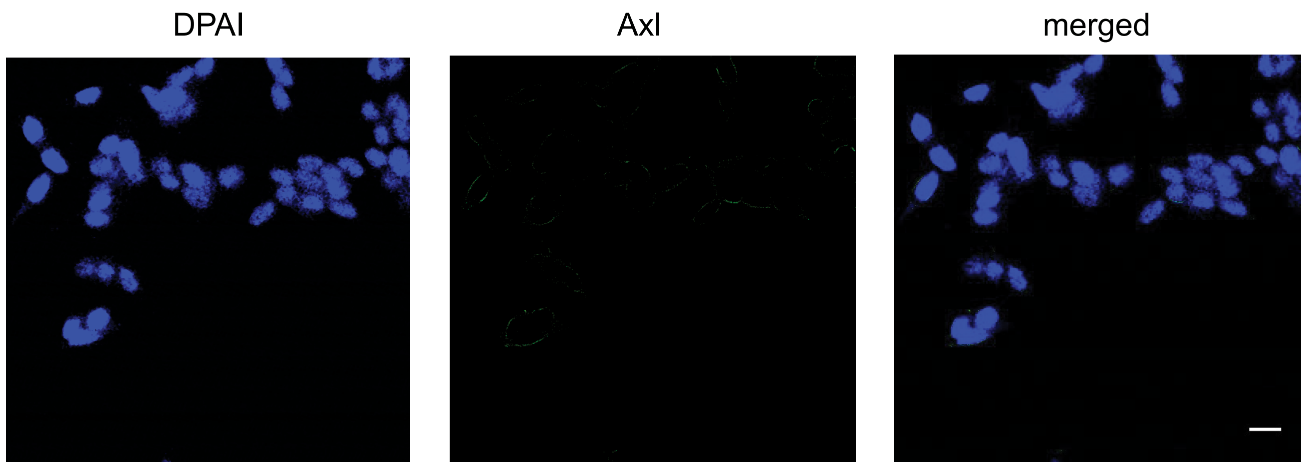

Figure S5. Expression of Axl is absent in differentiated SH-SY5Y cells. Cells were treated with 2 days of retinoic acid and infected with ZIKV-UG, followed by fixation and staining at 48 hpi. Staining for nuclei by DAPI is shown in dark blue. No immunofluorescent signal corresponding to Axl is seen (middle panel). The images shown are representative images taken from 3 independent experiments, with observation of a minimum of 10 fields under both low (10X) and high (40X and 63X) magnification per experiment.
